# Investigation of Pyrophosphates KYP_2_O_7_Co-Doped with Lanthanide Ions Useful for Theranostics

**DOI:** 10.3390/nano9111597

**Published:** 2019-11-11

**Authors:** Adam Watras, Marta Wujczyk, Michael Roecken, Katarzyna Kucharczyk, Krzysztof Marycz, Rafal J. Wiglusz

**Affiliations:** 1 Institute of Low Temperature and Structure Research PAS, Okolna 2 str. 50-422 Wroclaw, Poland; m.wujczyk@intibs.pl (M.W.); r.wiglusz@intibs.pl (R.J.W.); 2 Faculty of Veterinary Medicine, Equine Clinic-Equine Surgery, Justus-Liebig-University, 35392 Giessen, Germany; Michael.Roecken@vetmed.uni-giessen.de; 3 International Institute of Translational Medicine, Jesionowa 11, Malin, 55-114 Wisznia Mala, Poland; kucharczyk.katarzyna@o2.pl (K.K.); krzysztof.marycz@upwr.edu.pl (K.M.); 4 Department of Experimental Biology, Wroclaw University of Environmental and Life Sciences, 50-375 Wroclaw, Poland; 5 Collegium Medicum, Cardinal Stefan Wyszyński University (UKSW), Woycickiego 1/3, 01-938 Warsaw, Poland

**Keywords:** diphosphates, up-conversion, theranostics

## Abstract

Diphosphate compounds (KYP_2_O_7_) co-doped with Yb^3+^ and Er^3+^ ions were obtained by one step urea assisted combustion synthesis. The experimental parameters of synthesis were optimized using an experimental design approach related to co-dopants concentration and heattreatment as well as annealing time. The obtained materials were studied with theinitial requirements showing appropriate morphological (X-Ray Diffraction (XRD), Scanning Electron Microscopy (SEM)) and spectroscopic properties (emission, luminescence kinetics). Moreover, the effect of Er^3+^ and Yb^3+^ ions doped KYP_2_O_7_ on morphology, proliferative and metabolic activity and apoptosis in MC3T3-E1 osteoblast cell line and 4B12osteoclasts cell line was investigated. Furthermore, the expression of the common pro-osteogenic markers in MC3T3-E1 osteoblast as well as osteoclastogenesis related markers in 4B12 osteoclasts was evaluated. The extensive in vitro studies showed that KYP_2_O_7_ doped with 1 mol% Er^3+^ and 20 mol% Yb^3+^ ions positively affected the MC3T3-E1 and 4B12 cells activity without triggering their apoptosis. Moreover, it was shown that an activation of mTOR and Pi3k signaling pathways with 1 mol% Er^3+^, 20 mol% Yb^3+^: KYP_2_O_7_ can promote the MC3T3-E1 cells expression of late osteogenic markers including RUNX and BMP-2. The obtained data shed a promising light for KYP_2_O_7_ doped with Er^3+^ and Yb^3+^ ions as a potential factors improving bone fracture healing as well as in bioimaging (so-called in theranostics).

## 1. Introduction

In recent years, much attention has been paid to rare earth phosphate phosphors due to their appealing features, such as chemical stability and diversity in crystallographic structure [1]. The phosphates could be used as a matrix for doping with optically active ions, such as the rare earth metals. Potential application of the rare earth phosphates could be related to such areas as: cell bioimaging [2,3,4], light-emitting diodes [5,6,7,8], solar cells [9,10,11] as well as regenerative medicine.

Potassium yttrium(III) diphosphate(V) KYP_2_O_7_ is a polymorphic compound. Depending on the annealing temperature so-called the low temperature phase (β-KYP_2_O_7_) or the high-temperature phase (α-KYP_2_O_7_) could be obtained. On the basis of ionic radius ratio (*r*_K_^+^/*r*_Y_^3+^ = 1.68) value, polymorphism of KYP_2_O_7_ can be explained [12]. Three different synthesis routs were published for the KYP_2_O_7_: solid state reaction [13], one step urea-combustion synthesis [14] and boric acid flux method [15]. According to our knowledge the modern luminescent material KYP_2_O_7_ has never been employed as a matrix for investigation of up-conversion processes in biomedical applications.

Recently, tissue engineering together with regenerative medicine has become amore and more powerful tool in the field of bone regeneration as well as theranostics [16,17,18,19]. There are serious requirements for developing a strategy that could improve bone fracture regeneration, especially for elderly patients suffering from osteoporosis [20,21]. Bone fracture naturally involves two opposite processes, i.e., osteogenesis and osteoclastogenesis. The balance between these two processes ensures a new bone formation and finally bone regeneration. In the osteogenesis process the bone tissue formation is directly mediated by osteoblasts. This process is regulated on gene expression level by several transcripts including collagen type II, bone morphegenic protein 2 (BMP2), osteocalcin (OCL), osteopontin (OPN) and alkaline phospotase (ALP). The dynamic process thatis bone formation is mediated by several signaling pathways including mammalian target of rapamycin (mTOR) and phosphoinositide 3-kinase (Pi3k) regulating osteoblastogenesis and osteoclastogenesis. The activation of osteoclastis required for proper bone shaping and providing access to bone-stored minerals [22]. Thus, the induction of osteoblasts as well osteoclast activity and maintaining proper balance between them ensures proper bone remodeling and fracture regeneration, since over activity of osteoclast will lead to bone resorption. This phenomenon is well-known for several disorders including osteoporosis. The ability to improve osteoblasts viability with simultaneous inhibition of osteoclastogenesis seems to be a real challenge for novel materials. Moreover, the modern materials that serve additional functionality i.e., bioluminescence, which allows visualizing regenerative processes in a non-invasive way, are strongly required. The bioluminescent agents including Er^3+^ and Yb^3+^ besides their physical functions might additionally promote osteoblast activity, which can serve as their additional benefit. 

In this paper, samples of KYP_2_O_7_ co-doped with Er^3+^ and Yb^3+^ ions, were obtained using the one step urea-combustion method. Moreover, the spectroscopic investigation of occurring up-conversion processes into KYP_2_O_7_ matrix doped with Er^3+^ and Yb^3+^ ions was presented. Furthermore, the effects of KYP_2_O_7_ doped with 1 mol% Er^3+^ and 20 mol% Yb^3+^ on MC3T3-E1 osteoblasts and 4B12 osteoclasts were investigated paying special attention to viability, apoptosis, mitochondrial activity as well as an expression of common osteogenic and osteoclastogenesis related markers on mRNA levels.

## 2. Materials and Methods 

The *x* mol% Er^3+^, *y* mol% Yb^3+^:KYP_2_O_7_ (where *x* = 0.25, 0.50, 0.75, 1, 2, 5; *y* = 1, 2, 5, 10, 15, 20) powders were obtained by one step urea assisted combustion synthesis on the grounds of the synthesis route described elsewhere by R. Pązik et. al [14]. Reactants weight was calculated in stoichiometric manner with an exception to 10% excess for K_2_CO_3_·1.5H_2_O as well as to 20% excess of CH_4_N_2_O in reference to metal cations. The raw materials used for the synthesis purpose are: Y_2_O_3_ (Alfa Aesar GmbH & Co KG, Karlsruhe, Germany, 99.99%), Er_2_O_3_ (Alfa Aesar GmbH & Co KG, 99.99%), Yb_2_O_3_ (Alfa Aesar GmbH & Co KG, 99.99%), K_2_CO_3_·1.5H_2_O (Chempur, Piekary Slaskie, Poland, 99.0%), CH_4_N_2_O (PPH “POCh” S.A. Gliwice, Poland, 99.5%), (NH_4_)_2_HPO_4_ (Carl Roth GmbH + Co. KG, Karlsruhe, Germany, 99.999%), HNO_3_ (POCH S.A., Gliwice, Poland, 65%, ultrapure). Each of the final mixtures was dried for 24 h at 90 °C, later annealed at series of temperature ranging from 600 °C up to 800 °C for 4, 8 and 12 h.

The X-ray diffraction patterns were obtained by the use of X’Pert Pro PANalytical diffractometer (Cu, Kα1: 1.54060 Å) (Malvern Panalytical Ltd., Malvern, UK) in a 2θ range of 10°–50°, with a scan rate of 1.3°/min for 30 min at a room temperature. Investigation of morphology was performed using scanning microscope, specifically the FEI Nova NanoSEM 230 microscope (FEI Company, Hillsboro, OR, USA) equipped with the EDS spectrometer (EDAX PegasusXM4). Hydrodynamic size of the particles dispersed in water was determined by the use of dynamic light scattering technique supported by Zetasizer Nano-ZS (Malvern Panalytical Ltd., Malvern, UK) that is equipped with the He-Ne 633 nm laser (see Figure S1). Also, zeta potential was distinguished (see Figure S1). The emission spectra, as well as power dependence functions were recorded using the laser diode (λ_exc_ = 980 nm), with regulated power ranging from 0 to 4 W (Changchun New Industries Optoelectronics Tech. Co. Ltd., Jilin, China). For measurements the KG5 Schott filter was applied and as an optical detector the Hamamatsu PMA-12 photonic multichannel analyzer (Hamamatsu Photonics K.K., Hamamatsu City, Japan) was used. Furthermore, the obtained emission spectra are the result of averaged measurements, where the fixed parameters are the exposure time (200 ms) and the cumulative amount of measurements (15). Decay curves were collected using the tunable Ti:Sapphire laser (LOTIS TII, Minsk, Belarus) (λ_exc_ = 980 nm) pumped by the second harmonic of the YAG:Nd^3+^ pulse laser (ƒ = 10 Hz, *t* < 10 ns).

Mice osteoblasts MC3T3-E1 and osteoclasts 4B12 were used in this study. The cells were cultured in Minimum Essential Medium (MEM) Alpha w/o ascorbic acid (Gibco A10490-01) supplemented with 10% of Fetal Bovine Serum (FBS) (SigmaAldrich, Lenexa, KS, USA) with addition of 1% Penicillin/Streptomycin (P/S) (Sigma Aldrich, USA). In turn 4B12 cells were cultured in EMEM Alpha (Sigma M0200) supplemented with 10% of FBS and 30% of calvaria-derived stromal cell conditioned media (CSCM) without addition of antibiotic. The MC3T3-E1 were cultivated at 80% of confluence and they were passaged every 5 days by enzymatic dissociation using Trypsin-EDTA solution (SigmaAldrich, Saint Louis, MO, USA). Cells were incubated at 37 °C in a humidified atmosphere with 5% CO_2_.

Cell metabolic activity was measured by means of TOX-8 resazurin-based method using in vitro toxicology assay kit. MC3T3-E1 and 4B12 cells were plated into 96-well plates (3 × 10^3^ cells per well) in 4 replicates. Next metabolic activity of MC3T3-E1 were measured when cells were exposed to *x* = 10, 15 and 20 mol% of KYP_2_O_7_:1 mol% Er^3+^, *x* Yb^3+^, which was incorporated into the culture medium. For examination, compound was diluted in phosphate-buffered saline (PBS). First 10 mg of compound was suspended in 1 mL of PBS. Next this solution was added directly to cell culture medium in the proper concentration. The MC3T3-E1 and 4B12 cells were cultured in the presence and absence of tested materials for 120 h. After 24 and 120 h, culture medium was replaced with 10% solution of resazurin in fresh complete medium and incubated at 37 °C for 2h in CO_2_ cell culture incubator. Reduction of the dye was measured spectrophotometrically at a wavelength of 600 and 690 nm reference length (Epoch, Biotek, Bad Friedrichshall, Germany).

For analysis of genes expression, MC3T3-E1 and 4B12 cells were cultured 120 h onto KYP_2_O_7_:1 mol% Er^3+^, 20 mol% Yb^3+^. Then, cells were lysed in TRI Reagent and next total RNA was isolated using phenol-chloroform method described previously by Chomczynski and Sacchi [23]. To perform cDNA synthesis gDNA was digested with RNase-free (ThermoScientific^TM^, Whaltam, MA, USA), DNase I and next cDNA was synthesized using Tetro cDNA Synthesis Kit (Bioline, London, UK). qRT-PCR was performed using CFX Connect^TM^ Real-Time PCR Detection System (Bio-Rad, Hercules, CA, USA) for gene expression analysis. Reaction mixture contained 1 μL of cDNA in a total volume of 10 μL using SensiFAST SYBR & Fluorescein Kit (Bioline, London, UK). The concentration of primers in each reaction was equal to 500 nM; primer sequences used in individual reactions are listed in Table 1. The algorithm used for quantitative expression of the investigated genes was performed using the 2^−ΔΔCT^ method in relation to housekeeping gene (GAPDH).

To visualize theactin cytoskeleton and location of mitochondria the epifluorescent microscope (Olympus Fluoview FV1200, Tokyo, Japan) was used. Cells cultured onto KYP_2_O_7_:1 mol% Er^3+^, 20 mol% Yb^3+^ were stained with PhalloidinAtto 488 staining for F-actin visualization. For this purpose, the cells were fixed in 4% paraformaldehyde (PFA) (Sigma Aldrich) for 45 min at RT, then washed with phosphate-buffered saline (PBS) (SigmaAldrich) three times and permeabilized using 0.3% Tween 20 (SigmaAldrich) in PBS for 15 min. For nuclei visualization PhalloidinAtto 488 in PBS (dilution 1:700) (SigmaAldrich) staining for 45 min was performed. Obtained pictures were analyzed using ImageJ software 1.51j version (NIH, Bethesda, MD, USA). For the visualization of the mitochondria network, staining with the MitoRed was performed. For this purpose the culture medium was removed and cells were washed twice with PBS. After that the culture medium with MitoRed (1:1000) was added to cells in an amount equivalent to 350 μL per well. Cells were incubated for 30 min, after that they were washed three times with PBS. Later 4% PFA was added in an amount equal to 300 μL per well for 45 min, then cells were washed three times with PBS and put on DAPI. To visualize the cells morphology the contrast phase photos was taken (Zeiss, Oberkochen, Germany).

## 3. Results

### 3.1. Structural Analysis

**Figure 1:**
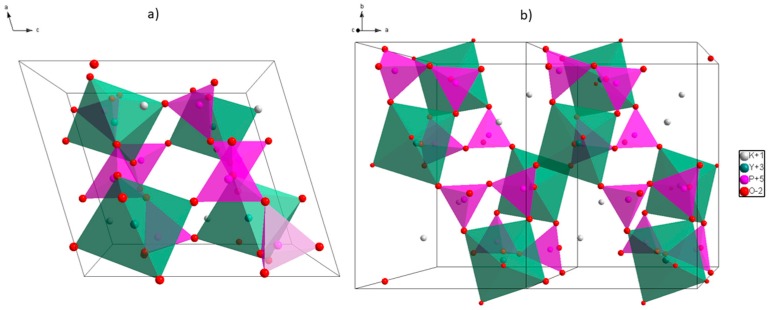
Projection of the β-KYP_2_O_7_ unit cell (**a**) and super cell (**b**) indicating the Y^3+^ and P^5+^ coordination polyhedra.

The β-KYP_2_O_7_ crystallizes in monoclinic system that belongs to the *P2_1_/c* space group and the α-phase crystallizes in orthorhombic system that can be assigned to the *Cmcm* space group. In the matrix Y^3+^ ions are substituted by the selected optically active ions RE^3+^, herein meaning Er^3+^ and Yb^3+^ Figure 1.

**Figure 2:**
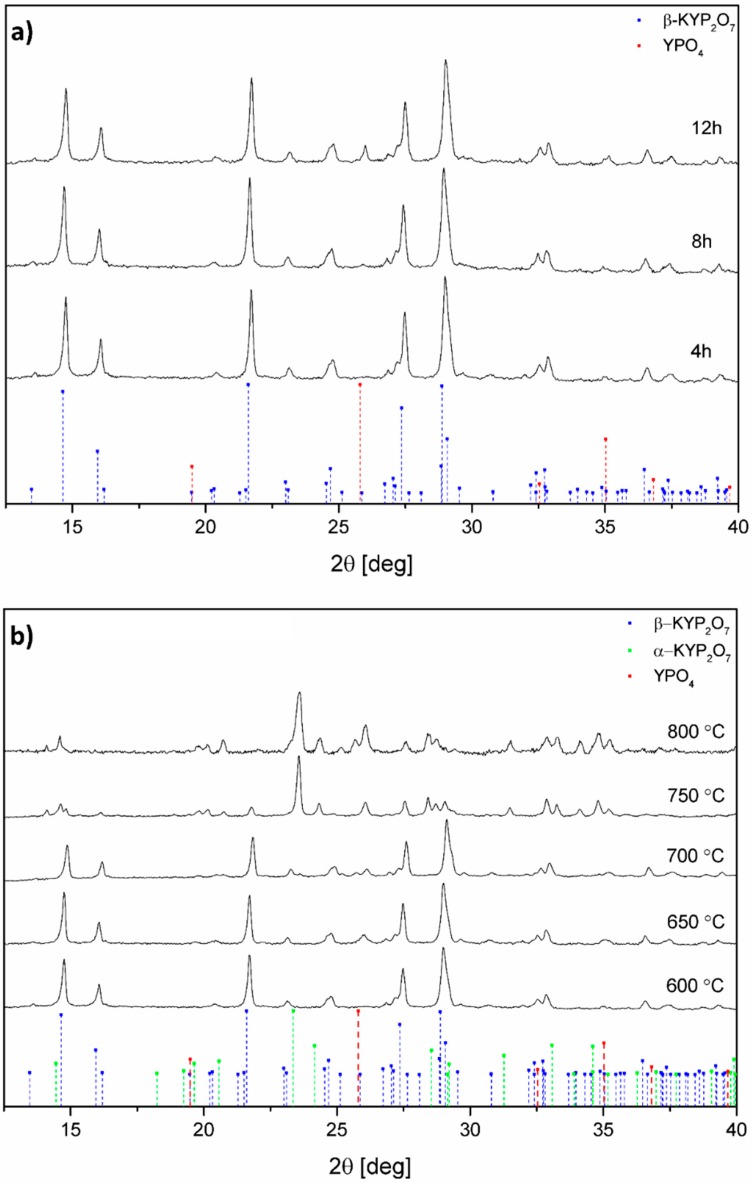
Representative XRD patterns of 1 mol% Er^3+^, 1 mol% Yb^3+^:KYP_2_O_7_ annealed at 600 °C for 4, 8 and 12 h (**a**) as well as annealed at 600–800 °C for 4 h (**b**).

The X-ray diffraction patterns were collected for all of the samples (see Figure 2 and Figures S2 and S3). Independently of the dopant concentration and the annealing time, each collected X-ray diffraction pattern shows a match to the theoretical pattern no. 160190 from ICSD. Up to the annealing temperature of 700 °C the sample crystalizes in the low temperature phase β-KYP_2_O_7_ (see Figure 2b).

Above the annealing temperature of 750 °C, the high-temperature phase α-KYP_2_O_7_ can be observed, matched with the pattern no. 75171 from ICSD. In the case of the annealing temperature from 750 to 800 °C, a decrease in the amount of β-KYP_2_O_7_ phase can be observed in favor of the high-temperature α-KYP_2_O_7_ phase. XRD patterns show presence of the YPO_4_ phase. Although the peaks from the YPO_4_ phase (ICSD no. 184543) overlap with β-KYP_2_O_7_, one could be noticed that this additional phase manifests itself in an increased intensity of certain peaks, when compared to the β-KYP_2_O_7_ theoretical pattern.

**Figure 3:**
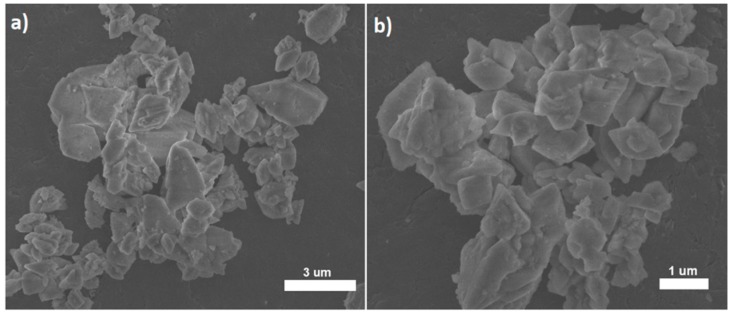
Representative SEM images of the 1 mol% Er^3+^, 1 mol% Yb^3+^:KYP_2_O_7_, annealed at 600 °C for 12 h with different magnifications (**a**) with 3 um scale bar and (**b**) with 1 um scale bar.

Obtained, representative SEM images of the1 mol% Er^3+^, 1 mol% Yb^3+^: KYP_2_O_7_ material, annealed at 600 °C for 12 h have been shown in Figure 3 in two different magnifications.

### 3.2. Luminescence Properties

**Figure 4:**
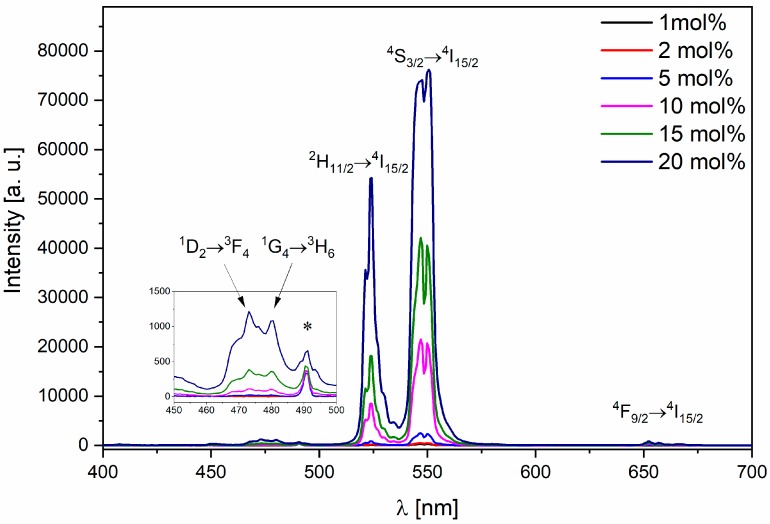
Representative emission spectra of KYP_2_O_7_ doped with *x* mol% Yb^3+^ ions and co-doped with 1 mol% Er^3+^under the excitation wavelength λ = 980 nm, *P* = 1.56 W, heat-treated at 650 °C for 12 h.

The emission spectra were measured at room temperature (300 K) with excitation wavelength λ_exc_ = 980 nm of the continuous wave (CW) laser power of 1.56 W (see Figure 4 and Figures S4–S6). Measurements were carried out for the samples annealed at two temperatures, 600 and 650 °C for 12 h with varying content of the co-dopants. Each of the spectrum consists of five bands, three of them can be assigned as Er^3+^ transitions ^2^H_11/2_→^4^I_15/2_, ^4^S_3/2_→^4^I_15/2_, ^4^F_9/2_ →^4^I_15/2_ observed respectively at 522, 540 and 650 nm. Samples annealed at 650 °C globally show more intense emission in comparison to those annealed at 600 °C. Within samples with varying content of Er^3+^ ions and fixed at 15 mol% concentration of Yb^3+^ ions, the highest emission intensity shows the one doped as follows: 1 mol% Er^3+^, 15 mol% Yb^3+^. Among all samples with varying content of Yb^3+^ and concentration of Er^3+^ fixed at 1 mol%, the most intense emission can be ascribed to the sample with 20 mol% of Yb^3+^, regardless of the annealing temperature.

In emission spectra, additional bands at 470 and 480 nm can be observed. These bands can be assigned to transitions occurring in Tm^3+^ ions, respectively ^1^D_2_→^3^F_4_ and ^1^G_4_→^3^H_6_. 

In addition, in the emission spectra a band at the wavelength of 490 nm is noticed and marked with asterisks in Figure 4 and Figures S4–S6. The emission corresponds to the second harmonic generation (SHG) from the excitation source, which is a diode laser λ_exc_ = 980 nm.

**Figure 5:**
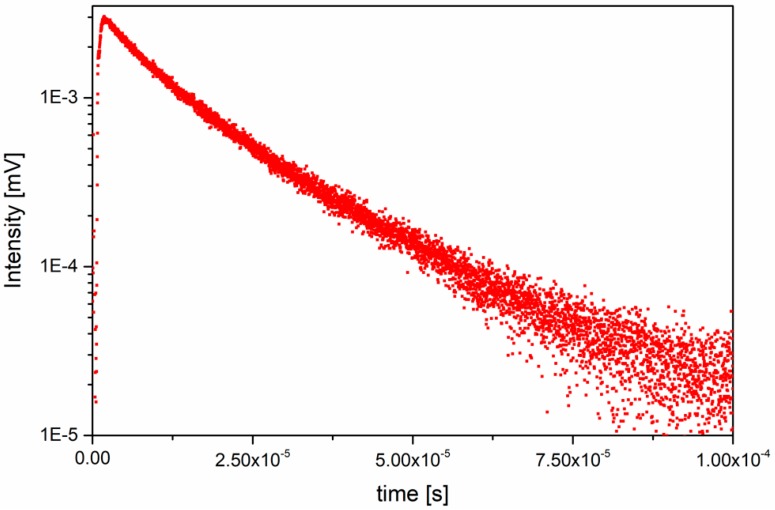
Decay time measured for 15 mol% Yb^3+^, 1 mol% Er^3+^:β-KYP_2_O_7_ annealed at 650 °C for 12 h.

Decay profiles were measured for the ^4^S_3/2_→^4^I_15/2_ transition prominent at 547 nm wavelength. Measurements were employed at room temperature (300 K) for the samples with varying concentration of the dopants, annealed 650 °C for 12 h. Each of decay curves was fitted with the double exponential function in Figure 5. 

Values of the two decay times, the fast component τ_1_ and the slow component τ_2_, are listed in Table 2. Due to the low emission intensity determination of the decay times for the samples with the lowest concentration of dopants were impossible. In case of the fixed value of erbium concentration, the increase in concentration of ytterbium is followed by the elongation of decay times. For higher concentrations of the dopants, reduction in the decay times can be observed. In addition, there is a correlation between the decay times and the intensity of the emission spectra. Those samples with the high intensity of emission can be assigned to the long decay times as well. 

### 3.3. Metabolic Activity, Morphology and Apoptosis of MC3T3-E1 Osteoblasts and 4B12 Osteoclast Cultured onto KYP_2_O_7_:1 mol% Er^3+^, x mol% Yb^3+^

**Figure 6:**
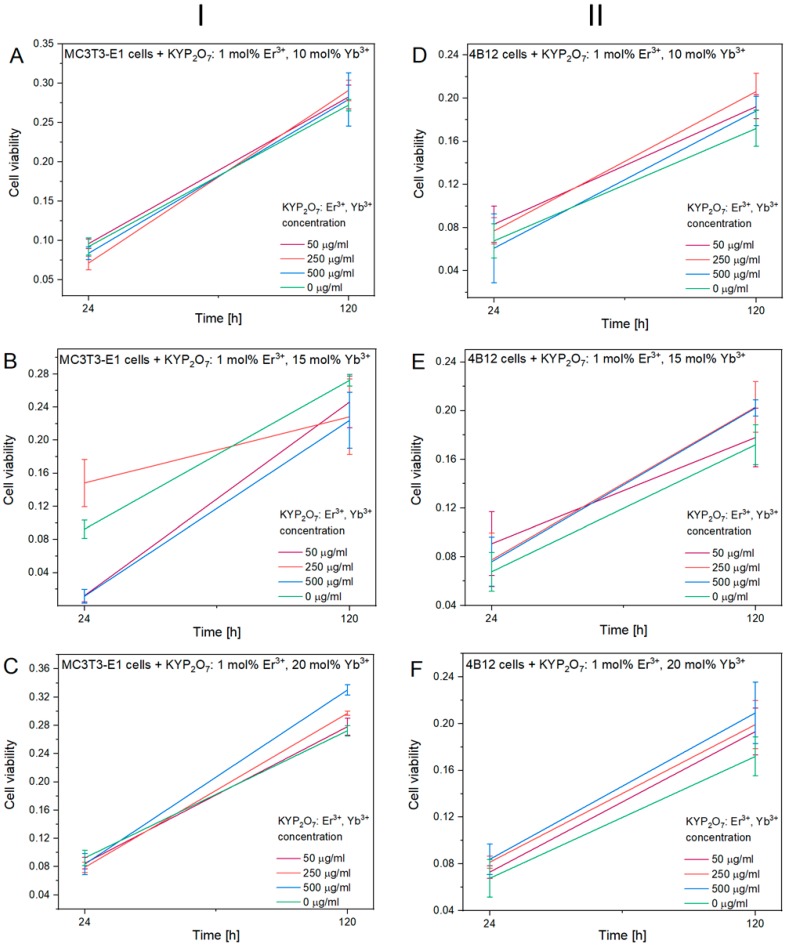
The viability and proliferative activity of MC3T3-E1osteoblasts(I) and 4B12osteoclast(II) cultured onto (**A**,**D**) 1 mol% Er^3+^, 10 mol% Yb^3+^:KYP_2_O_7_; (**B,E**) 1 mol% Er^3+^, 15 mol% Yb^3+^:KYP_2_O_7_ and (**C**,**F**) 1 mol% Er^3+^, 20 mol% Yb^3+^:KYP_2_O_7_addition after 24 and 120 h.

The viability and proliferative rate analysis of osteoblasts cultured onto KYP_2_O_7_:1 mol% Er^3+^, 20 mol% Yb^3+^ materials showed their beneficial effect on MC3T3-E1 number of cells (Figure 6). The highest proliferative activity of MC3T3-E1 cells was observed when they were exposed to KYP_2_O_7_: 1 mol% Er^3+^doped with 20 mol% of Yb^3+^in dose 500 µg/mL. Incorporation of 20 mol% Yb^3+^ in KYP_2_O_7_: 1 mol% Er^3+^ resulted in constant metabolic improvement through 120 h culture test. Similar effect was observed in 4B12 osteoclast cells, which reached the highest metabolic activity after 120 h, when cultured onto KYP_2_O_7_:1 mol% Er^3+^ doped with 20 mol% Yb^3+^ in dose 500 µg/mL. On the basis of mentioned results KYP_2_O_7_:1 mol% Er^3+^doped with 20 mol% of Yb^3+^in dose 500 µg/mL was used in further experiments.

**Figure 7:**
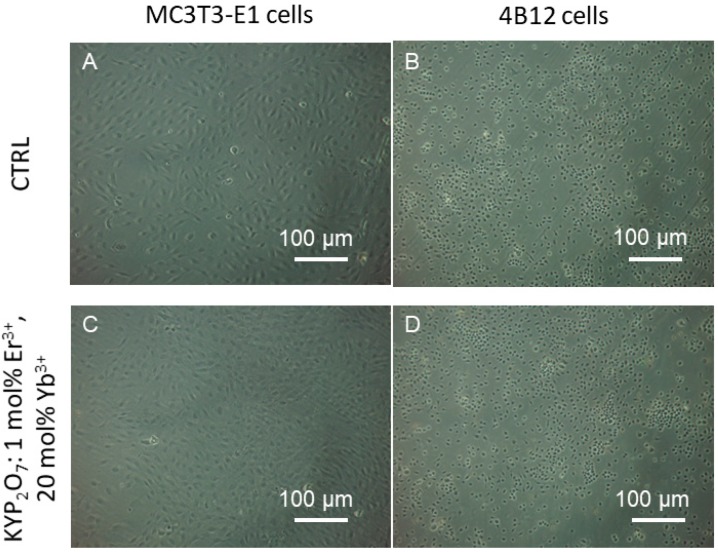
The MC3T3-E1 osteoblasts (**A**) and 4B12 osteoclasts (**B**) morphology visualized in control cells and in cells cultured with KYP_2_O_7_ doped with 1 mol% Er^3+^ ions co-doped with 20 mol% Yb^3+^ ions in dose 500 µg/mL in MC3T3 cells (**C**) and 4B12 cells (**D**) by contrast phase microscope. Magnification ×100, scale bars: 100 μm.

**Figure 8:**
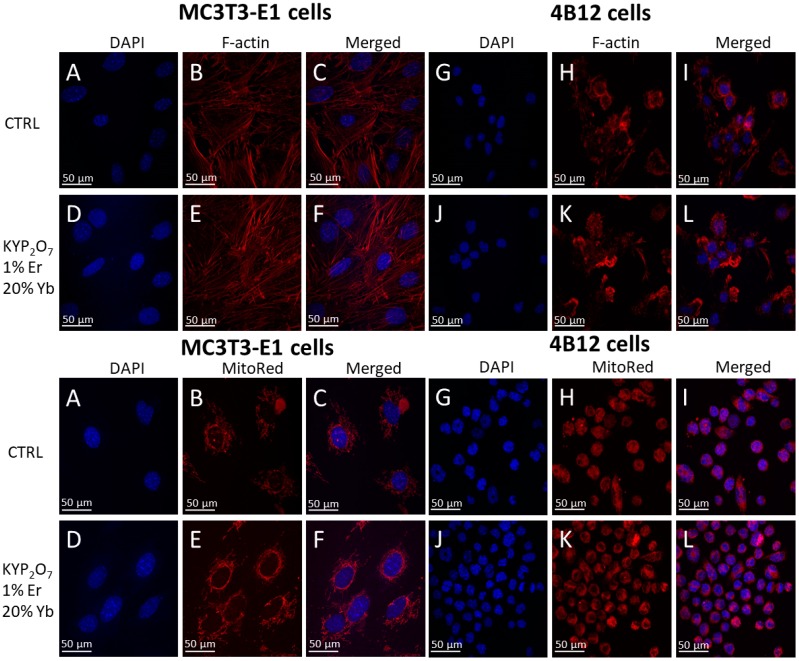
The F-actin, DAPI and MitoRed staining. (**A**,**B**,**C)** in upper graphs presented F-actin and DAPI staining of MC3T3-E1 cells; (**D,E,F**) showed F-actin and DAPI staining of MC3T3-E1 cells with investigated material KYP_2_O_7_ doped with 1 mol% Er^3+^ ions co-doped with 20 mol% Yb^3+^ ions in dose 500 µg/mL; (**G,H,I**) in upper graphs presented F-actin and DAPI staining off 4B12 cells; (**J,K,L**) showed F-actin and DAPI staining of 4B12 cells with investigated material KYP_2_O_7_ doped with 1 mol% Er^3+^ ions co-doped with 20 mol% Yb^3+^ ions in dose 500 µg/mL; (**A,B,C**) in lower graphs presented MitoRed and DAPI staining off MC3T3-E1cells; (**D,E,F**) showed MitoRed and DAPI staining of MC3T3-E1 cells with investigated material KYP_2_O_7_ doped with 1 mol% Er^3+^ ions co-doped with 20 mol% Yb^3+^ ions in dose 500 µg/mL; (**G,H,I**) in lower graphs MitoRed and DAPI staining off 4B12 cells; (**J,K,L**) showed MitoRed and DAPI staining of 4B12 cells with investigated material KYP_2_O_7_ doped with 1 mol% Er^3+^ ions co-doped with 20 mol% Yb^3+^ ions in dose 500 µg/mL. Scale bars presented in the images obtained using epifluroescent microscope were equal 50 μm.

**Figure 9:**
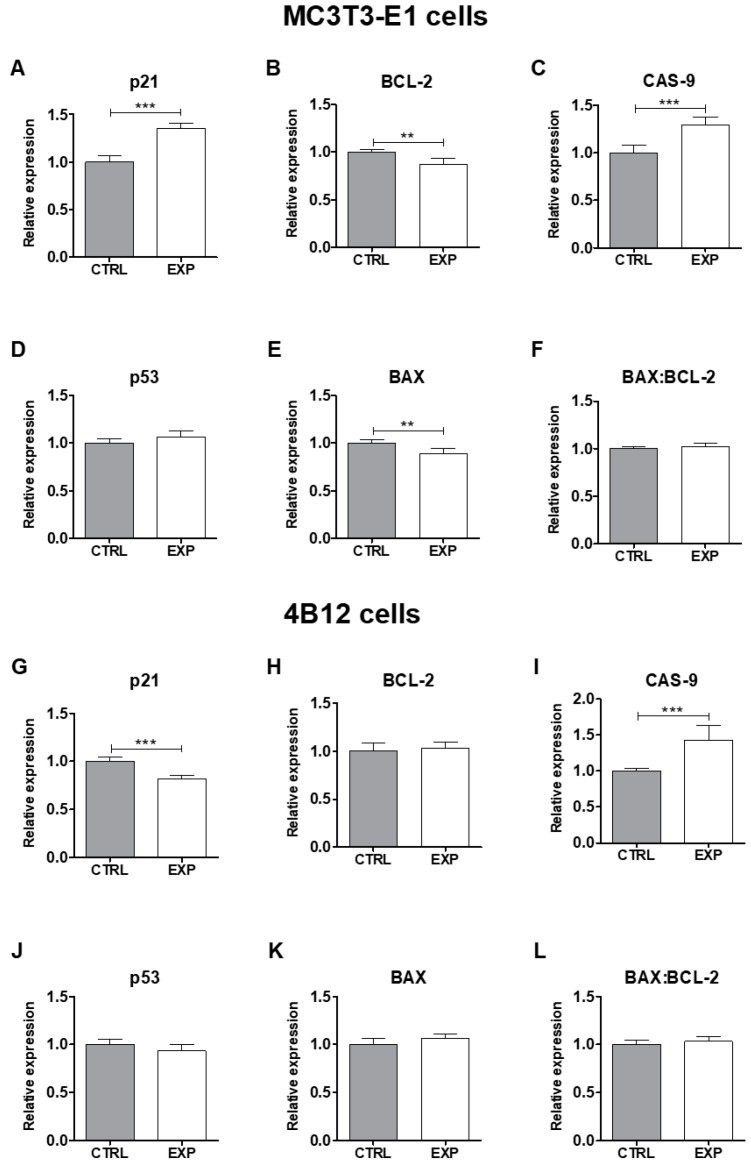
Evaluation of apoptosis in MC3T3-E1 osteoblasts and 4B12 osteoclast. To evaluate apoptosis in cells, the expression of (**A,G**) p21, (**B,H**) Bcl-2, (**C,I**) CAS-9, (**D,J**) p53 (**E,K**) BAX was analyzed. The (**I,L**) BAX:BCL-2 ratio was calculated using relative expression values of both BCL-2 and BAX.

For analysis of cells morphology the contrast phase pictures was taken (Figure 7). It was found that Yb^3+^ in the 500 ug/mL dosage positively affects morphology of both osteoblasts as well as osteoclasts. For analysis of the mitochondrial and actin network of MC3T3-E1 osteoblasts and 4B12 osteoclast, KYP_2_O_7_:1 mol% Er^3+^ co-doped with 20 mol% Yb^3+^ was proceeded. The creation of abundant actin network was observed in MC3T3-E1 osteoblasts when cultured onto KYP_2_O_7_:1 mol% Er^3+^ co-doped with 20 mol% Yb^3+^ when compared to the control group. The cells presented typical for osteoblast round-like shape morphology with a well visible nuclei. Moreover, cells communicated with each other and created a well-developed cell-to-cell network, as shown by the well-developed actin network (Figure 8). Actin network also testifies of increased adhesion of osteoblast when cultured onto KYP_2_O_7_:1 mol% Er^3+^, 20 mol% Yb^3+^. In the case of the osteoclasts, we also observed more developed cytoskeleton and actin staining showed well develop actin network when the cells were cultured with KYP_2_O_7_:1 mol% Er^3+^, 20 mol% Yb^3+^. Mitochondrial staining revealed that in both cells type i.e., osteoblasts and osteoclasts, dense network around nuclei was created, when the cells were cultured onto KYP_2_O_7_:1 mol% Er^3+^, 20 mol% Yb^3+^. It might suggest thata 500 μg/mL dose significantly promotes mitochondrial biogenesis, which resulted in creation of well-developed mitochondrial network (Figure 8). Moreover, it seems that this dose induces slight apoptosis in MC3T3-E1 osteoblasts while no prominent effect was observed in osteoclast cells. Incorporation of KYP_2_O_7_:1 mol% Er^3+^, 20 mol% Yb^3+^ into osteoblasts culture resulted in significant up regulation of p21 and Cas-9 mRNA level since BAX transcript was significantly down regulated (Figure 9). It was found that p21 transcript was considerably down regulated in osteoclast cells when cultured onto KYP_2_O_7_:1 mol% Er^3+^, 20 mol% Yb^3+^ in comparison for control culture.

### 3.4. Expression of Osteogenic and Osteclastogenic Markers in MC3T3-E1 Osteoblasts and 4B12 Osteoclast Cultured onto KYP_2_O_7_: 1 mol% Er^3+^, 20 mol% Yb^3+^in Relation to mTOR and Pi3K Pathway

**Figure 10:**
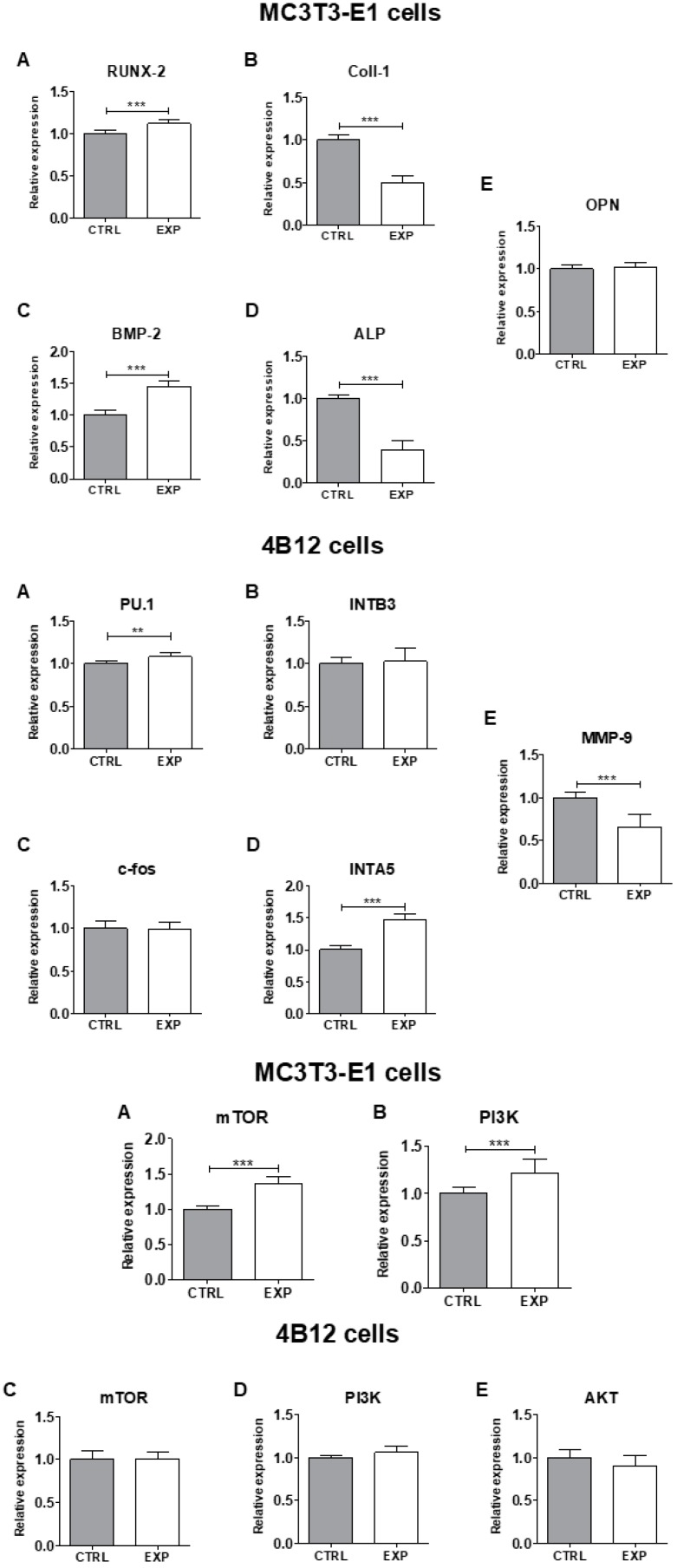
Comparison of the expression levels of osteogenesis-related genes using quantitative real-time PCR analysis. The expression of (**A**) RUNX-2, (**B**) Coll-1, (**C**) BMP2, (**D**) ALP, and (**E**) OPN in MC3T3-E1 osteoblasts and the expression of (**A**) PU.1, (**B**) INTB3, (**C**) c-fos, (**D**)INTA5 and (**E**) MMP-9 in 4B12 osteoclast cultured onto KYP_2_O_7_ doped with 1 mol% Er^3+^, 20 mol% Yb^3+^ ions. In lower graphs the expression of (**A,C**) mTOR and (**B,D**) PI3K and (**E**) AKT in MC3T3-E1 cells and 4B12 cells was presented.

Evaluation of the expression of pro-osteogenic markers on mRNA level in MC3T3-E1 osteoblasts showed beneficial effect of KYP_2_O_7_:1 mol% Er^3+^, 20 mol% Yb^3+^ material on osteogenesis process (Figure 10). It was found that KYP_2_O_7_:1 mol% Er^3+^, 20 mol% Yb^3+^ promotes in MC3T3-E1 cells expression of RUNX-2 as well as BMP-2 mRNA level, since reduces expression of Coll-1 and ALP transcripts. In turn, it was observed that KYP_2_O_7_:1 mol% Er^3+^, 20 mol% Yb^3+^ promotes in 4B12 osteoclast expression of PU. 1, which is involved in regulation of beta(3) integrin expression during osteoclast differentiation. Moreover, elevated expression of INTA5 in 4B12 osteoclasts was observed.

The elevated expression of both mTOR as well as Pi3K in MC3T3-E1 osteoblasts was observed when cultured onto KYP_2_O_7_:1 mol% Er^3+^, 20 mol% Yb^3+^ material in comparison to the control group (Figure 10). Moreover, in 4B12 osteoclast a significant reduction of MMP-9 expression was found together with up regulation of INTA5 transcript. There were no significant differences between mTOR and Pi3K expression in 4B12 osteoclast exposed to KYP_2_O_7_:1 mol% Er^3+^, 20 mol% Yb^3+^ and control. 

## 4. Discussion

Obtained X-ray patterns for samples annealed at variety of temperature show decreasing presence of β-KYP_2_O_7_ phase in favor of α-KYP_2_O_7_ phase beginning at 750 °C. Optimal heat treatment parameters were set to be: 600 °C, 12 h and 650 °C, 12 h. Given parameters allow gratifying emission properties, shown in latter section, for up-conversion process characterization. Further spectroscopic and biological analysis were employed for the samples annealed with aforementioned parameters. Our observations are in accordance with literature data. It has already been proven, by us and others that α-KYP_2_O_7_ phase dominates over β-KYP_2_O_7_ one above 700 °C [14,15]. In reference to annealing time and doping level of β-KYP_2_O_7_ no reports were found. However, for up-conversion processes in different matrices doping level of Yb^3+^ and Er^3+^ ions similar to concentrations stated as optimal in this paper [24,25]. Size and morphology of the KYP_2_O_7_:Er^3+^, Yb^3+^ powders were estimated using SEM microscopy, in Figure 3 shown are agglomerates (≈ 3 µm) of elongated submicron particles with the shape of flat plates.

On the basis of the emission spectra, the highest intensity emission band can be assigned to the sample with concentration ratio of 1 mol% Er^3+^ and 20 mol% Yb^3+^, when annealed at 650 °C for 12 h. For concentrations higher than 1 mol% Er^3+^ a decrease in emission band intensity can be observed, due to concentration quenching of activators’ emission [26]. Hence, the optimal doping concentration was chosen to be 1 mol% Er^3+^. The samples heavily doped with Yb^3+^ ions, where the above-mentioned phenomenon is not observed, show a monotonic increasement of intensity within analyzed concentration range (1–20 mol% Yb^3+^). Therefore, the optimal doping concentration was chosen to be 20 mol% Yb^3+^. Decay times of analyzed samples show direct correlation with emission spectra. Emission’s intensity increasement is followed with decay time elongation. The samples exhibiting concentration quenching deviate from the mentioned trend and consequently reduction in decay time is being observed. Lengthening of the decay time might refer to an occurrence of energy transfer between upconverting ions [27].

Measurements of power dependence (PD) (see Figure S7), shown as a double-logarithmic function of emission intensity versus laser pump power, allow for estimating several absorbed photons vital for up-conversion process occurrence [28]. Results assert a two-photon nature of the ^2^H_11/2_→^4^I_15/2_ and the ^4^S_3/2_→^4^I_15/2_ transitions at λ = 522–540 nm with *n* values varying from 1.8 to 2.0. Therefore, the anti-Stokes emission may occur via two routes: Energy Transfer Up-conversion (ETU) or Excited State Absorption (ESA). ETU is the most efficient one out of all UC processes, as a resemblance to the full resonance is the closest [29].These UC processes are not easy to distinguish by power dependence, owing to the fact that *n* value equals 2 for all cases. Short decay times for samples with minor content of co-dopants may indicate dominance of ESA, while highly doped samples might be favoring ETU, due to their longer decay times. Occurrence of the UC processes can be distinguished also with presence of arise and further prolongation of rise time in decay time function.

Weak emission intensity of the ^4^F_9/2_→^4^I_15/2_ transition at λ = 650 nm shows that metastable ^4^F_9/2_ state is not being favorably populated. PD measurements, for aforementioned transition, show the *n* value equal to 1.0–1.3, letting us believe that the ^4^F_9/2_→^4^I_15/2_ transition is influenced by non-linear, nonradiative process, such as cross relaxation. 

Presence of transitions from Tm^3+^ seen in emission spectra, may stem from contamination of reactants, herein especially erbium oxide. It is a well-known fact that Tm^3+^ ion can play a role of an activator in UC processes, similarly to Er^3+^ ions, if matrix is co-doped with Yb^3+^ ions. Hence, thulium ions compete as an activator with erbium ions. 

The materials dedicated for bone fracture regeneration require specific characteristics including stimulation of bone formation processes as well as inducing matrix formation. The phosphates are well-known for their pro-osteogenic ability; however, KYP_2_O_7_ doped with rare earths elements including Er^3+^ and Yb^3+^ ions were not previously investigated. In this study, we showed that KYP_2_O_7_ doped with 1 mol% of Er^3+^ and 20 mol% Yb^3+^in dose 500 µg/mL promotes osteoblasts metabolic activity and induces their highest proliferative potential. In previous research using nanometric hydroxyapatites doped with Er^3+^ we observed a similar effect; however on stem progenitor cells and olfactory ensheathing cells [30]. Moreover, we observed that KYP_2_O_7_:1 mol% Er^3+^, 20 mol% Yb^3+^ promotes also proliferative and metabolic activity of osteoclast. Furthermore, the cytoskeleton development including actin formation was noted in MC3T3-E1 osteoblasts as well as 4B12 osteoclasts when they were exposed for KYP_2_O_7_:1 mol% Er^3+^, 20 mol% Yb^3+^. The observed arrangement of actin fibers indicates about fully stretched of cells and this allows us to evaluate the material as biocompatible [31]. Additionally, we observed improved cell-to-cell contact and creation of a well-developed network suggesting beneficial effect of KYP_2_O_7_:1 mol% Er^3+^, 20 mol% Yb^3+^ on matrix formation. Interestingly, similar to osteoblasts, osteoclasts presented a well-developed cytoskeleton and actin network. The beneficial effect of KYP_2_O_7_:1 mol% Er^3+^, 20 mol% Yb^3+^ on osteoblasts activity might be associated with improved mitochondrial biogenesis and creation of dense mitochondrial network. The mitochondria morphology and especially their fission and fusion is one of the elements of assessment cells viability, senescence and metabolism [32]. We indicated that examined material improved mitochondria network and not causes their fission what evidence about positive influence of KYP_2_O_7_:1 mol% Er^3+^, 20 mol% Yb^3^ on cells viability. Together with improved mitochondrial function, we observed that KYP_2_O_7_:1 mol% Er^3+^, 20 mol% Yb^3+^ negatively affects expression of p21 and Cas-9 on mRNA level. Obtained data indicate on rather neutral role of KYP_2_O_7_:1 mol% Er^3+^, 20 mol% Yb^3+^ on osteoblasts apoptosis although significant down regulation of BAX transcripts was observed. Moreover, the appearance of nuclei, after staining with DAPI showed that KYP2O7:1 mol% Er^3+^, 20 mol% Yb^3+^ not implicates the chromatin condensation and DNA fragmentation, which indicates the lack of induce apoptosis by the examined material and well biocompatible of it [33]. What is more important, the beneficial effect of the material for pro-osteogenic genes expression including RUNX-2 as well as BMP-2 mRNA in MC3T3-E1 cells was observed. Interestingly, at the same time reduced expression of Coll-1 and ALP transcripts was noted. Obtained data clearly indicates on promotion of early markers of osteogenesis expression instead late markers expression. It suggests that KYP_2_O_7_:1 mol% Er^3+^, 20 mol% Yb^3+^ might exert a beneficial effect on bone mineralization process and matrix formation. Observed pro-osteogenic effect of KYP_2_O_7_:1 mol% Er^3+^, 20 mol% Yb^3+^ on MC3T3-E1 osteoblasts might be partially explained by the elevated expression of both mTOR as well as Pi3K signaling pathways. It was previously showed that both mTOR as well as Pi3K are positively associated with bone formation and bone remodeling effect [34]. What is more, we observed that KYP_2_O_7_:1 mol% Er^3+^, 20 mol% Yb^3+^ enhanced the expression of BMP-2 and mTOR in MC3T3 osteoblasts. That fact indicates on pro-osteogenic properties of fabricated material as interplay between these two protein was shown to modulate and enhance osteogenesis [35]. Moreover, is worth adding that KYP_2_O_7_:1 mol% Er^3+^, 20 mol% Yb^3+^decreased expression of MMP-9. The increased level of this metalloproteinase is typical for osteoporotic bones. So the fact that modulation of the amount of transcripts MMP-9 by KYP_2_O_7_: 1 mol% Er^3+^, 20 mol% Yb^3+^ affects the restoration of the balance between osteoblasts and osteoclasts in osteoporotic bones.

## 5. Conclusions

Optimization of the Potassium yttrium(III) diphosphate(V) synthesis parameters and a degree of doping was reached. Research shows a stable β-KYP_2_O_7_ crystallographic structure and gratifying spectroscopic properties, obtained by finding optimal synthesis conditions (such as annealing temperature, annealing time and degree of doping). The heating parameters of 600 and 650 °C as well as the heating time of 12 h were considered the best parameters of the synthesis process. Globally the most intense emission was obtained for samples co-doped with 1 mol% Er^3+^ and 20 mol% Yb^3+^ ions. In addition, the studies were carried out to consider KYP_2_O_7_ co-doped with erbium and ytterbium ions, as a future material used in biomedical applications, especially theranostics. Additionally, phosphate KYP_2_O_7_ doped with 1 mol% of Er^3+^ and 20 mol% Yb^3+^ positively affects MC3T3-E1 osteoblasts morphology, proliferative as well as metabolic activity. Although no positive effect in relation to apoptosis was found, KYP_2_O_7_:1 mol% Er^3+^, 20 mol% Yb^3+^ significantly promotes expression of early markers of osteogenesis via mTOR as well as Pi3K which sheds a promising light on that system as an agent promoting fracture bone regeneration. Moreover, observed inhibitory effect on osteoclastogenesis suggests the potential beneficial role of KYP_2_O_7_:1 mol% Er^3+^, 20 mol% Yb^3+^ in treatment of osteoclast related disorders. 

## Figures and Tables

**Table 1:** Sequences of primers used in qRT-PCR.

Gene	Primers (5’→3’)	Length of Amplicon	Accession No.
p53	F: AGTCACAGCACATGACGGAGG R: GGAGTCTTCCAGTGTGATGATGG	287	NM_001127233.1
BCL-2	F: GGATCCAGGATAACGGAGGC R: ATGCACCCAGAGTGATGCAG	141	NM_009741.5
BAX	F: AGGACGCATCCACCAAGAAGC R: GGTTCTGATCAGCTCGGGCA	251	NM_007527.3
p21	F: TGTTCCACACAGGAGCAAAG R: AACACGCTCCCAGACGTAGT	175	NM_001111099.2
Cas-9	F: CCGGTGGACATTGGTTCTGG R: GCCATCTCCATCAAAGCCGT	278	NM_001355176.1
GAPDH	F: TGCACCACCAACTGCTTAG R: GGATGCAGGGATGATGTTC	177	NM_001289726.1

F: forward; R: reverse; p53: tumor suppressor p53; BCl-2: B-cell lymphoma; BAX: Bcl-2 associated X protein; p21: cyclin dependent kinase inhibitor 1A; Cas-9: Caspase-9; GAPDH: Glyceraldehyde 3-phosphate dehydrogenase.

**Table 2:** Luminescence decay times for β-KYP_2_O_7_ samples annealed at 650 °C for 12 h.

Dopants Concentration		Decay Times	
Er^3+^ (mol%)	Yb^3+^ (mol%)	τ_1_ (µs)	τ_2_ (µs)
1	5	1.13	8.59
	10	5.14	17.56
	15	7.52	19.94
	20	6.96	18.85
0.50	15	0.91	6.73
0.75		5.85	19.90
1		7.52	19.94
2		5.92	12.06
5		3.26	6.25

## Supplementary Materials

The following are available online at https://www.mdpi.com/2079-4991/9/11/1597/s1, Figure S1: Results of the dynamic light scattering (DLS) expressed via z-average size parameter and zeta potential measurements for the representative sample KYP_2_O_7_:1 mol% Er^3+^, 1 mol% Yb^3+^ heat-treated at 650 °C for 12 h; Figure S2: XRD patterns of β-KYP_2_O_7_ annealed at 600 °C for 12 h with varying content of Yb^3+^ ions and fixed 1 mol% Er^3+^ (a) as well as with varying content of Er^3+^ and fixed 15 mol% Yb^3+^ (b); Figure S3: XRD patterns of β-KYP_2_O_7_ annealed at 650 °C for 12 h with varying content of Yb^3+^ ions and fixed 1 mol% Er^3+^ (a) as well as with varying content of Er^3+^ and fixed 15 mol% Yb^3+^ (b); Figure S4: Emission spectra of KYP_2_O_7_ doped with *x* mol% Yb^3+^ ions and co-doped with 1 mol% Er^3+^ under the excitation wavelength λ = 980 nm, annealed at 600 °C for 12 h; Figure S5: Emission spectra of KYP_2_O_7_ doped with x mol% Er^3+^ ions and co-doped with 15 mol% Yb^3+^ under the excitation wavelength λ = 980 nm, annealed at 600 °C for 12 h.; Figure S6: Emission spectra of KYP_2_O_7_ doped with *x* mol% Er^3+^ ions and co-doped with 15 mol% Yb^3+^ under the excitation wavelength λ = 980 nm, annealed at 650 °C for 12 h; Figure S7: Power dependence measurements of the ^4^F_9/2_→^4^I_15/2_ (a) and of the ^2^H_11/2_, ^4^S_3/2_→^4^I_15/2_ (b) for samples KYP_2_O_7_ annealed at 650 °C.
