# Supplementary material for: Investigation of Pyrophosphates KYP2O7Co-Doped with Lanthanide Ions Useful for Theranostics
Source: Nanomaterials (Basel). 2019 Nov 11;9(11):1597. doi: 10.3390/nano9111597 (PMC6915349; doi:10.3390/nano9111597)
Supplement: Supplementary file 1 [file nanomaterials-09-01597-s001.pdf]

Investigation of Pyrophosphates KYP_2O_7Co-Doped with Lanthanide Ions Useful for Theranostics

Adam Watras [1,*], Marta Wujczyk [1], Michael Roecken [2], Katarzyna Kucharczyk [3,4], Krzysztof Marycz [,3,4,5] and Rafal J. Wiglusz [1]

[1] Institute of Low Temperature and Structure Research PAS, Okolna 2 str. 50-422 Wroclaw, Poland; m.wujczyk@intibs.pl (M.W.); r.wiglusz@intibs.pl (R.J.W.)
[2] Faculty of Veterinary Medicine, Equine Clinic-Equine Surgery, Justus-Liebig-University, 35392 Giessen, Germany; Michael.Roecken@vetmed.uni-giessen.de
[3] International Institute of Translational Medicine, Jesionowa 11, Malin, 55-114 Wisznia Mala, Poland; kucharczyk.katarzyna@o2.pl (K.K); krzysztof.marycz@upwr.edu.pl (K.M.)
[4] Department of Experimental Biology, Wroclaw University of Environmental and Life Sciences, 50-375 Wroclaw, Poland
[5] Collegium Medicum, Cardinal Stefan Wyszyński University (UKSW), Woycickiego 1/3, 01-938 Warsaw, Poland
* Correspondence: a.watras@intibs.pl

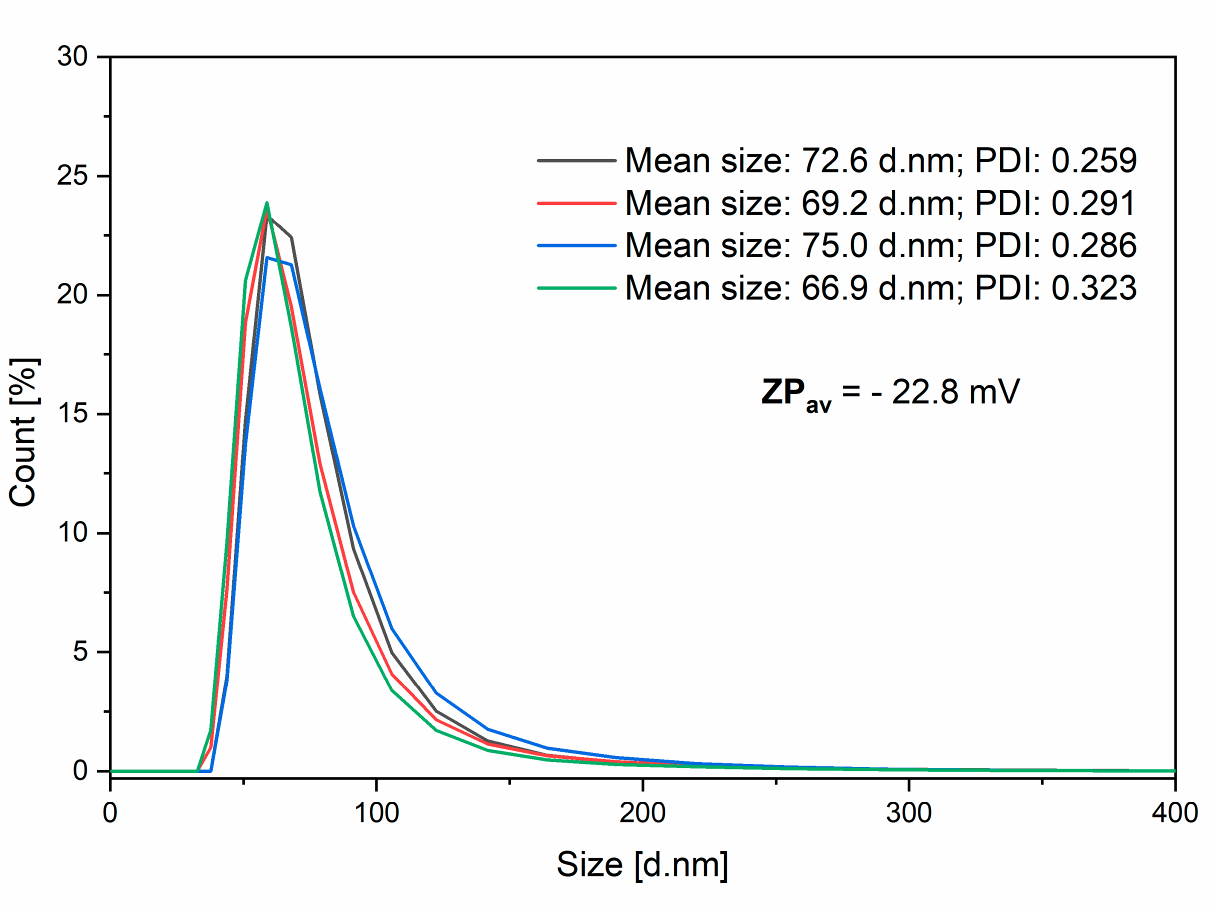

Figure S1. Results of the dynamic light scattering (DLS) expressed via z-average size parameter and zeta potential measurements (ZP_{av}) for the representative sample KYP_2O_7:1 mol% Er^{3+}, 1 mol% Yb^{3+} heat treated at 650 °C for 12 h.

Hydrodynamic size measurements have been performed by the use of Zetasizer Nano (Malvern). As a representative sample KYP_2O_7:1 mol% Er^{3+}, 1 mol% Yb^{3+} heat treated at 650 ℃ for 12 h has been used. Material was dispersed in water. Obtained size distribution results

present a mean size of particles around 70 d.nm. The mean size was distinguished, although the size distribution of the particles is not symmetric. Therefore a mode size for each measurement was determined and it equals 58.77 d.nm. Particles sizes are mostly distributed under 100 nm. A slight amount of larger particles is present, what can be stated on the basis of the presence of the "tail" occurring for sizes larger than 100 nm. Additionally average zeta potential has been measured and is equal to –22.8 mV for a representative sample KYP_2O_7:1 mol% Er^{3+}, 1 mol% Yb^{3+}.

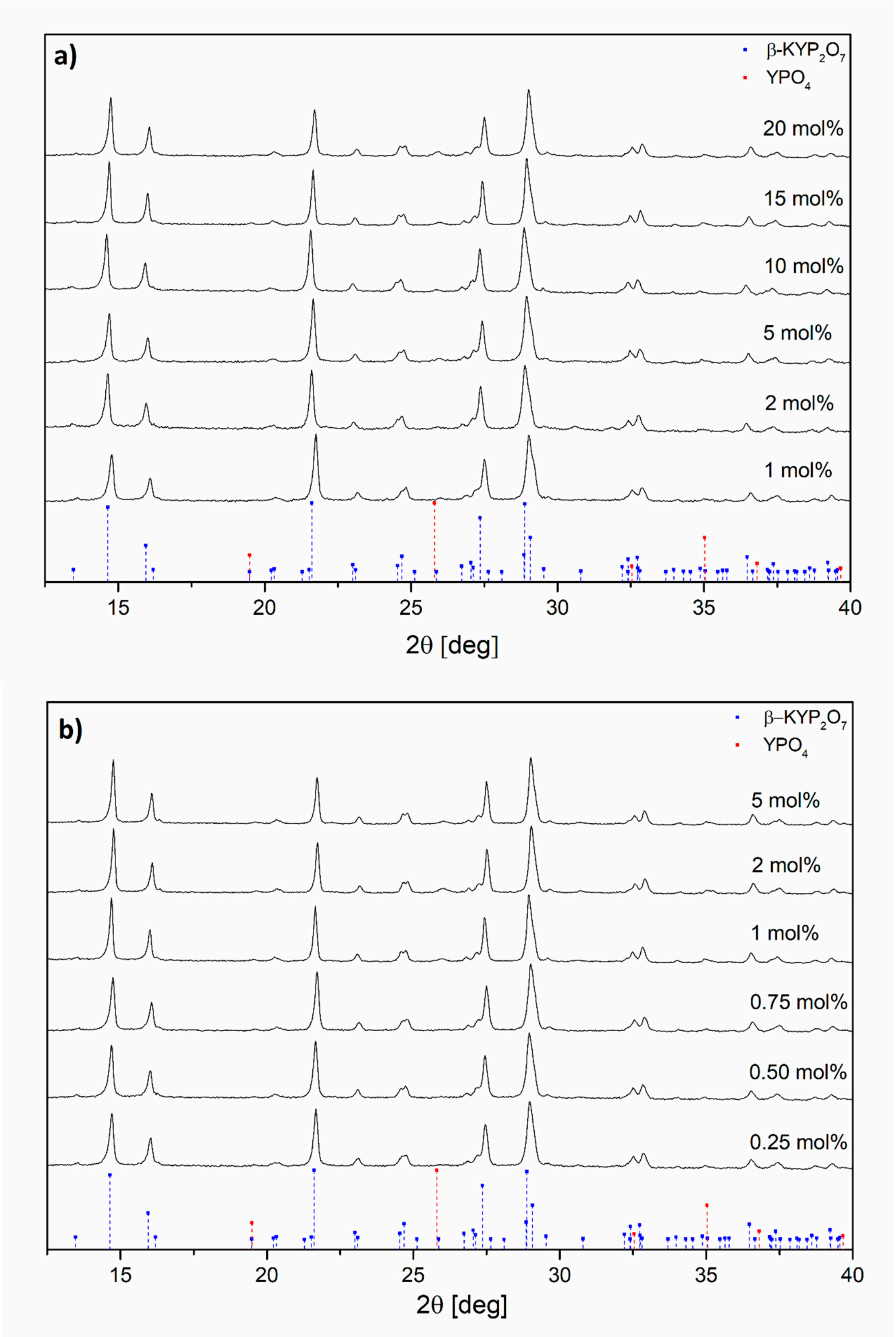

Figure S2. XRD patterns of β-KYP_2O_7 annealed at 600 °C for 12 h with varying content of Yb^{3+} ions and fixed 1 mol% Er^{3+} (**a**) as well as with varying content of Er^{3+} and fixed 15 mol% Yb^{3+} (**b**).

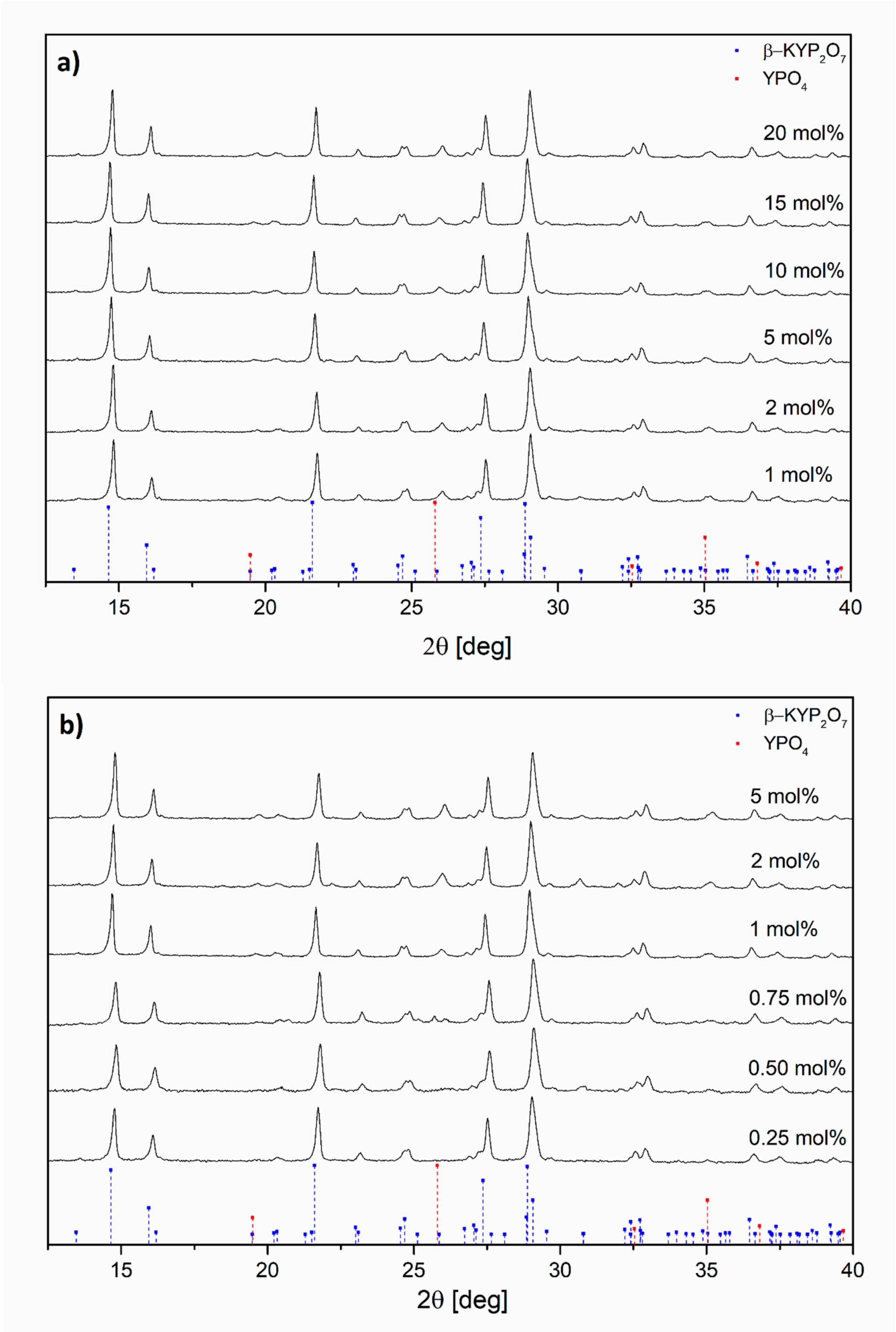

Figure S3. XRD patterns of β-KYP_2O_7 annealed at 650 °C for 12 hours with varying content of Yb^{3+} ions and fixed 1 mol% Er^{3+} (**a**) as well as with varying content of Er^{3+} and fixed 15 mol% Yb^{3+} (**b**).

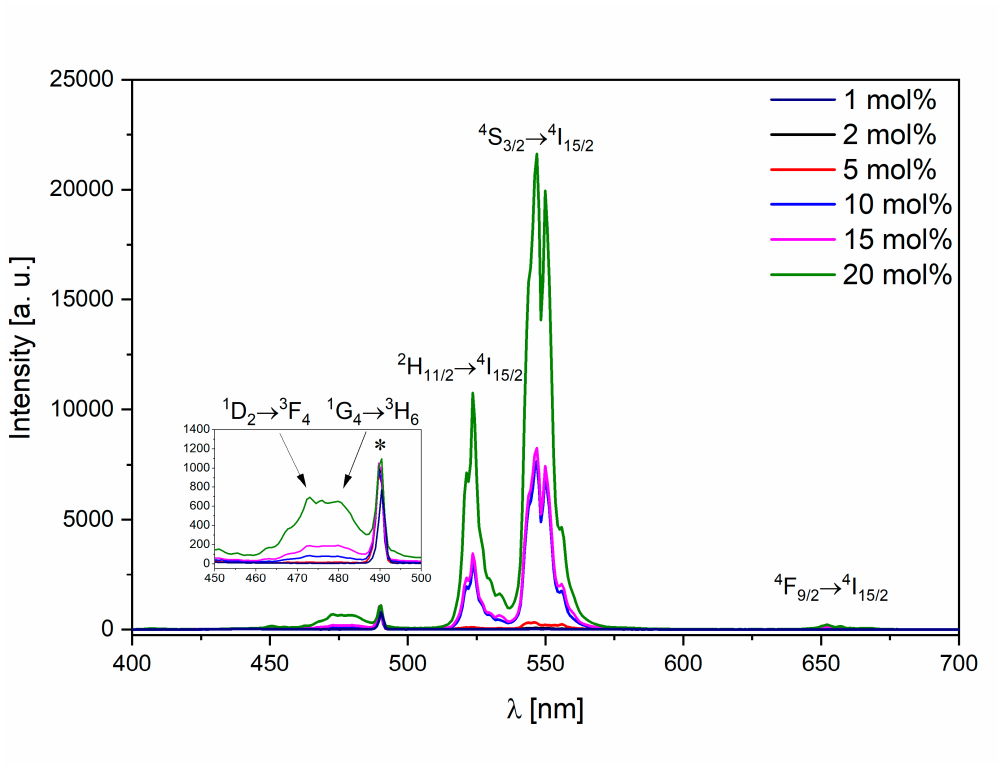

Figure S4. Emission spectra of KYP_2O_7 doped with *x* mol% Yb^{3+} ions and co-doped with 1 mol% Er^{3+} under the excitation wavelength λ = 980 nm, annealed at 600 °C for 12 h.

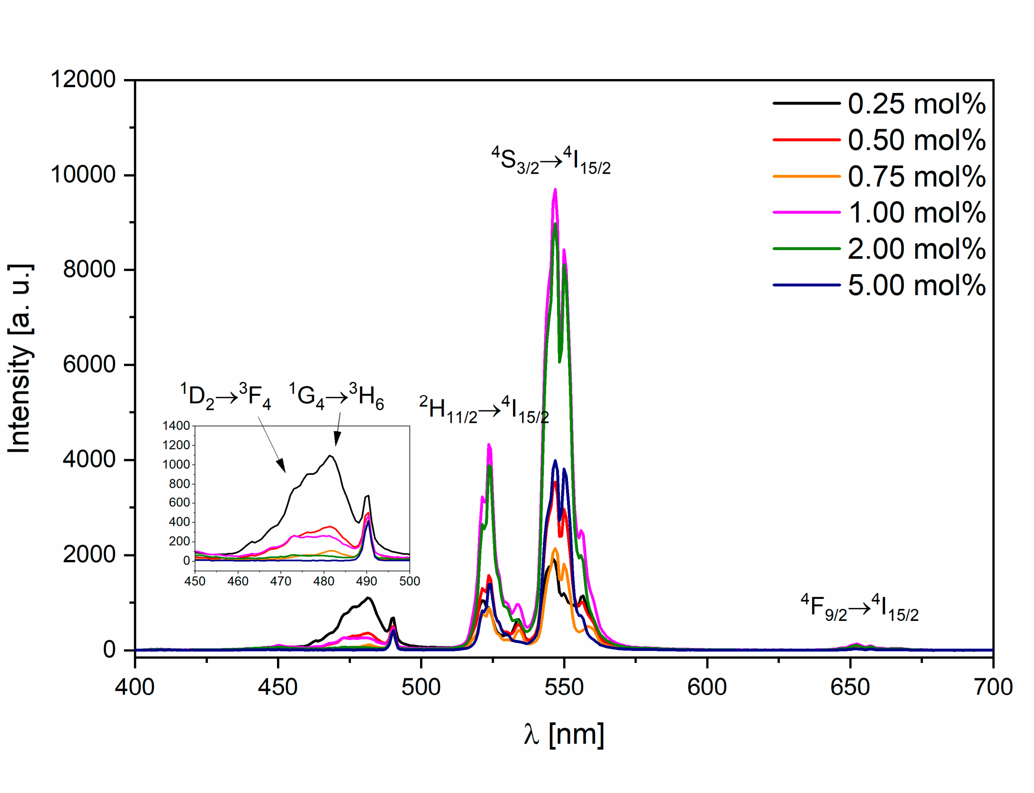

Figure S5. Emission spectra of KYP_2O_7 doped with *x* mol% Er^{3+} ions and co-doped with 15 mol% Yb^{3+} under the excitation wavelength λ = 980 nm, annealed at 600 °C for 12 h.

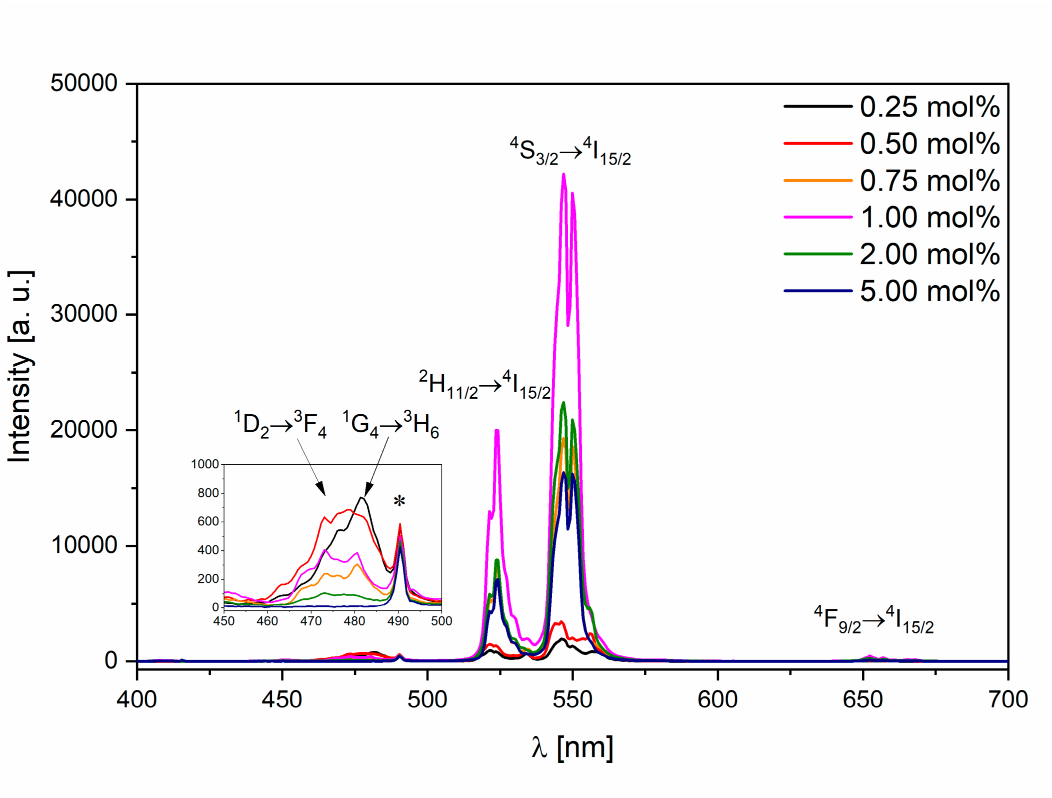

Figure S6. Emission spectra of KYP_2O_7 doped with x mol% Er^{3+} ions and co-doped with 15 mol% Yb^{3+} under the excitation wavelength λ = 980 nm, annealed at 650 °C for 12 h.

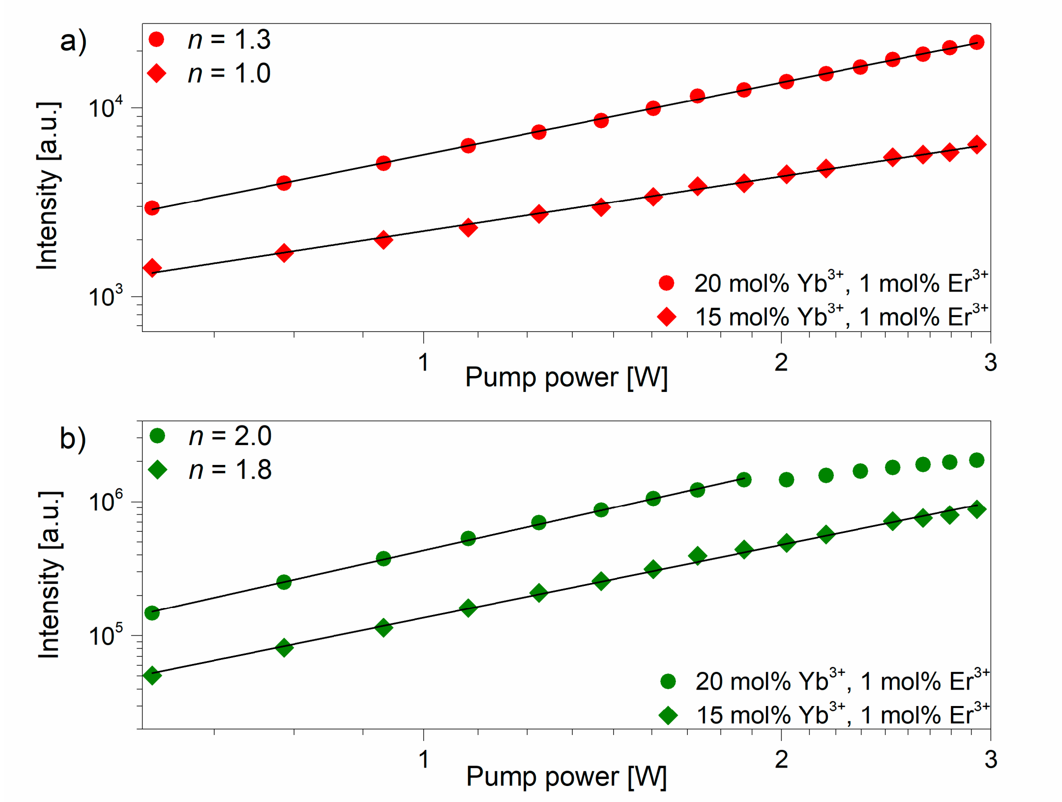

Figure S7. Power dependence measurements of the $^4F_{9/2}\rightarrow{}^4I_{15/2}$ (**a**) and of the $^2H_{11/2}$, $^4S_{3/2}\rightarrow{}^4I_{15/2}$ (**b**) for samples KYP_2O_7 annealed at 650 °C.
